# Molecular complexity of intraductal carcinoma of the prostate

**DOI:** 10.1002/cam4.6939

**Published:** 2024-02-20

**Authors:** Sha Zhu, Nanwei Xu, Hao Zeng

**Affiliations:** ^1^ Department of Urology, Institute of Urology, West China Hospital Sichuan University Chengdu China

**Keywords:** DNA damage and repair, Intraductal carcinoma of the prostate, MED12, molecular characteristics, MYC, NCOR2, prostate acinar adenocarcinoma, PTEN, RB1, SChLAP1, targeted therapy, TMPRSS2‐ERG fusion, TP53

## Abstract

Intraductal carcinoma of the prostate (IDC‐P) is an aggressive subtype of prostate cancer characterized by the growth of tumor cells within the prostate ducts. It is often found alongside invasive carcinoma and is associated with poor prognosis. Understanding the molecular mechanisms driving IDC‐P is crucial for improved diagnosis, prognosis, and treatment strategies. This review summarizes the molecular characteristics of IDC‐P and their prognostic indications, comparing them to conventional prostate acinar adenocarcinoma, to gain insights into its unique behavior and identify potential therapeutic targets.

## 1 INTRODUCTION

Intraductal carcinoma of the prostate (IDC‐P) represents a highly aggressive subtype of prostate cancer characterized by the growth of tumor cells inside the ducts and acinus of the prostate gland. This particular subtype is frequently observed alongside conventional prostate acinar adenocarcinoma (PAC) and is known to be associated with an unfavorable prognosis. Consequently, IDC‐P has emerged as a valuable phenotypic marker for identifying cases with a high risk of lethal disease progression.

As our understanding of cancer advances, it becomes increasingly evident that comprehending the molecular mechanisms driving each pathological subtype is crucial. This necessity holds especially true for IDC‐P due to its aggressive nature and its correlation with adverse patient outcomes. Therefore, gaining deeper insights into the molecular pathways and genetic perturbations underlying the development and progression of IDC‐P becomes imperative for the development of more effective diagnostic, prognostic, and therapeutic approaches against this deadly form of prostate cancer.

This review aims to provide a comprehensive exploration of the molecular characteristics of IDC‐P, shedding light on its distinct molecular features compared to invasive carcinoma. The clinical behavior of IDC‐P is believed to ultimately stem from its unique molecular features, which warrants further investigation.

This thorough analysis of current research findings reveals several aberrant genes and pathways associated with IDC‐P, unveiling key molecular alterations that contribute to its aggressive phenotype. These aberrations include dysregulation of oncogenes (more specifically, *MYC* and *ERG*) and tumor suppressors (including *TP53*, *RB1*, and *PTEN*), highlighting the disrupted balance between pro‐oncogenic and tumor‐suppressive signaling pathways. Additionally, DNA damage and repair machinery, crucial for maintaining genomic stability, appear to be dysregulated in IDC‐P. Furthermore, distinctive mutation patterns within the *androgen receptor (AR)* pathway, a vital driver of prostate cancer, have been observed in IDC‐P. This suggests that IDC‐P may possess unique mechanisms of androgen signaling and responsiveness. Transcription factors, such as *MED12*, and noncoding RNA molecules, like *SChLAP1*, have also emerged as noteworthy players in IDC‐P, potentially contributing to its aggressive behavior through their regulatory roles.

While these molecular aberrations likely contribute to the observed behavior of IDC‐P to some extent, comprehensive study designs are still needed to address fundamental questions. Understanding the origins of IDC‐P, the driving factors behind its progression and aggressiveness, and identifying potential therapeutic targets are crucial areas for future research. By conducting in‐depth examinations of the molecular aspects of IDC‐P, we can pave the way for improved strategies in the diagnosis, prognosis, and tailored treatment of this aggressive form of prostate cancer. A summary of the important molecular findings on IDC‐P is shown in Table 1.

**TABLE 1:** Summary of the important molecular findings on intraductal carcinoma of the prostate (IDC‐P).

Main findings	References
TP53, RB1, PTEN, and MYC	
Loss of heterozygosity (LOH) is more common in IDC‐P than in invasive cancer.	[1, 2]
LOH of *TP53, RB1*, and *PTEN* is more frequently found in IDC‐P than in invasive cancer.	[2]
Cribriform and intraductal carcinoma (CR/IDC) shows more percentage of genome altered (PGA) and somatic copy number alterations (CNA), including loss of *PTEN*, gain of *MYC*, and point mutations in *TP53*.	[3, 4, 5, 6, 7, 8]
Tumors with IDC‐P show higher homologous recombination deficiency scores, which is correlated with *MYC* mutations, *TP53* mutations and HRR pathway mutations in certain subgroups. Ref. 9 also shows E2F targets enriched in tumors with IDC‐P.	[6, 9, 10]
*MED12L/MED12* amplification is enriched in *BRCA2*‐mutant prostate cancer with IDC‐P, particularly in cases associated with a poor prognosis. *MYC* amplifications is enriched in *BRCA2*‐mutant prostate cancer with IDC‐P.	[11]
*TP53* mutation was linked to worse prognosis in patients with IDC‐P.	[12]
Isolated IDC‐P shows high percentage of activating oncogenic driver mutations in *MAPK* and *PI3K* pathway genes.	[13]
DNA damage and repair	
In Patient‐derived xenografts (PDXs), people found a link between IDC‐P and *BRCA2* variant. PDXs from tumors with *BRCA2* mutations had more IDC‐P cases than those with sporadic PCa. *BRCA2* carriers with IDC‐P had worse survival rates compared to those without IDC‐P.	[14]
Dysregulation of DNA repair genes and DNA damage sensing genes in IDC‐P was found in recurrent or metastatic prostate cancer patients.	[4, 7, 15]
IDC‐P tumors exhibit a higher occurrence of mismatch repair gene (MMR) aberrations, despite their general rarity in prostate cancer	[16]
The *BRCA2* mutation rate was much higher in IDC‐P patients compared to those without IDC‐P. The link between IDC‐P and DDR mutations holds true in Chinese patients, and it seems that Chinese IDC‐P patients also have more *CDK12* mutations.	[12]
NCOR2	
Patients with IDC‐P have been reported to be insensitive to ADT.	[17, 18, 19]
No big difference in *AR* mutation rates was found between tumors with and without IDC‐P. However, IDC‐P tumors had a significant increase in nuclear receptor corepressor 2 (*NCOR2*) histone deacetylase.	[12]
Decreased *NCOR2* expression is correlated with increased disease aggressiveness in prostate cancer. Knocking down *NCOR2* in cell line models induces the gene signature associated with neuroendocrine prostate cancer (NEPC).	[20]
MED12	
Increased methylation levels of the *APC* gene were found in IDC‐P lesions, which further contributes to the activation of the Wnt pathway.	[21]
Tumors with IDC‐P exhibited a higher prevalence of genomic and epigenomic dysregulation of *MED12*.	[11]
The prevalence of *MED12* mutations varies considerably among different prostate cancer study cohorts. Conflicting views exist regarding the functional consequences of *MED12* mutations in prostate cancer. The tumorigenic mechanisms associated with *MED12* mutations may be cancer specific.	[22, 23, 24]
TMPRSS2‐ERG fusion	
IDC‐P exhibits a higher prevalence of *TMPRSS2‐ERG* fusions than prostatic intraepithelial neoplasia (PIN).	[25, 26]
*TMPRSS2‐ERG* fusion rates are similar between IDC‐P and concurrent invasive carcinoma.	[27, 28, 29]
*TMPRSS2‐ERG* fusion is a relatively common event seen in prostate cancer.	[30, 31, 32, 33]
Some studies suggest significant associations between *TMPRSS2‐ERG* status and factors such as Gleason score, biochemical recurrence, and survival.	[31, 34, 35]
However, other studies have found no significant differences related to the presence of the gene fusion.	[32, 36, 37]
The combination of *PTEN* and *TP53* mutation status with the overexpression of *ERG* via *TMPRSS2‐ERG* fusion has shown predictive value for biochemical recurrence and survival.	[38]
SChLAP1	
The concept of “Nimbosus” was put forward in IDC‐P. *SChLAP1* is the only gene showing up with threefold higher expression in IDC‐P among over 25,000 genes.	[39]
The presence of numerous adverse molecular events in tumors with IDC‐P collectively contribute to the visibility on multiparametric magnetic resonance imaging and worse prognosis.	[40]

## 2 MORPHOLOGIC FEATURES AND PATHOLOGIC CHARACTERISTICS OF IDC‐P

Cribriform morphology in prostate cancer, observed in both invasive cribriform carcinoma (ICC) and IDC‐P, signifies an aggressive subtype linked to lethal progression.^41^, ^42^ ICC and IDC‐P commonly coexist, with 47% of ICC intermingling with IDC and 68% of IDC showing intermixing with ICC.^43^ Distinguishing IDC‐P from ICC often requires immunohistochemistry to identify the preservation of basal cells, which are observable in IDC‐P.^44^ Due to challenges in differentiation, IDC‐P and ICC are often assessed together in both research and clinical settings.

Architectural patterns in IDC‐P include micropapillary, loose cribriform, dense cribriform, and solid growth patterns.^45^, ^46^ Comedonecrosis could be observed in any of these patterns, but it is most frequently observed in dense cribriform and solid patterns.^47^ An issue yet to be resolved pertains to the grading of IDC‐P. The Genitourinary Pathology Society (GUPS)^48^ suggests excluding the IDC‐P component from the Gleason score (GS), whereas International Society of Urological Pathologists (ISUP)^49^ recommends incorporating this component into the GS. Both sets of experts concur that pure (isolated) IDC‐P without associated invasive component should not be graded.

Since the discovery and reporting of IDC‐P, the question of its origins has been a hot topic of controversy in both basic and clinical research. The fact that IDC‐P is accompanied with high‐grade PAC, in particular with ICC, has raised the question of whether there is an intrinsic evolutionary connection between them. Regarding this issue, we recently identified three evolutionary patterns between IDC‐P and concurrent PAC lesions: early divergent, late divergent, and clonally distant.^50^ These findings suggest bona fide heterogeneity between IDC‐P and PAC.

## 3 TP53, RB1, PTEN, AND MYC

In IDC‐P, widespread allelic loss is a common occurrence, likely attributed to its extensive degree of genomic instability.^1^, ^2^ This phenomenon results in the loss of critical tumor suppressor genes, including *TP53*, *RB1*, and *PTEN*, while also leading to the overexpression of oncogenes such as *MYC*.^2^, ^3^, ^4^, ^5^, ^6^, ^9^, ^10^, ^11^

TP53 and RB1 play critical roles in controlling cell cycle progression, with TP53 acting as a central hub for DNA damage response pathways.^51^ RB1 primarily regulates the G1 to S transition during the cell cycle,^52^ which is carried out by binding to and inhibiting the activity of the E2F family of transcription factors. Therefore, it is not surprising that IDC‐P exhibits enriched pathways associated with E2F targets.^9^

In addition, TP53, RB1, and MYC have been implicated in the development of neuroendocrine prostate cancer (NEPC), a subtype characterized by poor prognosis and resistance to standard treatments.^53^, ^54^ TP53 and RB1 have been reported to regulate the differentiation of prostate epithelial cells, and their loss can lead to NEPC development, possibly through the activation of neural crest stem cell programs.^54^, ^55^ MYC is a transcription factor that participates in the regulation of cell growth, proliferation, genomic instability, and differentiation, and its overexpression has been shown to contribute to the development of NEPC in mouse models.^10^, ^56^, ^57^ Compared to high‐grade prostatic intraepithelial neoplasia (HGPIN),^58^ a recognized precancerous lesion of prostate cancer, the loss of heterozygosity (LOH) involving both *TP53* and *RB1* genes is more frequently observed in IDC‐P.^2^ Furthermore, while *TP53* and *RB1* loss is uncommon in primary prostate cancer, it becomes more prevalent in metastatic castration‐resistant prostate cancer (mCRPC) (53.3% and 21%, respectively).^59^ In a study by Mateo et al.,^60^ clinically actionable genomic alterations were evaluated in 61 prostate cancer patients with matched samples from the same patient that are sensitive and resistant to androgen deprivation therapy (ADT). The analysis revealed notable differences between the two matched biopsies from the same patient, with increased *TP53*, *RB1*, and *PI3K/AKT* pathway alterations observed in mCRPC. Additionally, *RB1* loss in primary prostate cancer has been associated with poorer survival outcomes, and another study reported its correlation with the development into a castration‐resistant state and worse clinical outcomes.^61^ Interestingly, our analysis also indicated that *TP53* mutation was linked to a shorter castration‐resistant‐free survival (median 10.9 vs. 28.9 months, *p* = 0.026).^12^ Genomic aberrations in *TP53* and *RB1* have been associated with resistance to ADT,^62^ and they are commonly detected in nearly all NEPC tumors.^63^ Hence, similar to the evolution of NEPC, *TP53*, and *RB1* possess the potential to generate therapy resistance and contribute to disease progression through treatment selection pressures in IDC‐P.

*PTEN* inactivation represents another prevalent event in prostate cancer, displaying an even higher incidence in tumors featuring IDC‐P.^2^, ^3^, ^5^, ^6^, ^7^ The multifaceted role of PTEN as both a lipid and protein phosphatase exerts a broad range of effects on cellular processes, including cell polarity, motility, senescence, tumor microenvironment, and immune response.^64^ Moreover, besides *PTEN*, isolated IDC‐P cases lacking invasive carcinoma have shown an enrichment of driver mutations in other *MAPK/PI3K* genes, which are infrequent in conventional prostate cancer.^13^ This suggests a dysregulated network of kinases and phosphatases in IDC‐P, offering numerous potential targets for intervention. Notably, *PTEN* genomic deletion in localized prostate cancer has been linked to several adverse clinicopathological features such as higher GS and increased likelihood of extraprostatic extension.^65^ Several extensive studies have consistently established a clear association between PTEN loss and an elevated probability of biochemical recurrence following prostatectomy.^66^, ^67^, ^68^ Furthermore, PTEN has emerged as an independent predictor for metastasis, CRPC progression, and prostate cancer‐specific mortality.^69^, ^70^, ^71^ In a study^5^ that examined PTEN loss using immunohistochemical assays, a significant correlation was identified between PTEN loss and IDC‐P. Specifically, 69% of the total samples with IDC‐P exhibited PTEN loss compared to only 12% of PTEN intact samples. Furthermore, IDC‐P displayed the highest relative risk (4.99) for PTEN loss.^8^

Hence, the frequent dysfunction of these crucial cancer‐related genes in IDC‐P can contribute to uncontrolled cell cycle progression, genomic instability, as well as other yet undiscovered functions, ultimately fueling the aggressiveness of the tumor.

## 4 DNA DAMAGE AND REPAIR

Genomic instability in IDC‐P arises not only from the dysregulation of cell cycle checkpoints but also from the disruption of DNA damage and repair machinery (DDR). Extensive evidence links IDC‐P to dysregulated DNA damage and repair pathways.

The relation between IDC‐P and germline *BRCA2* variant was initially identified in patient‐derived xenografts (PDXs),^14^ and subsequent germline genetic testing in recurrent or metastatic prostate cancer patients further revealed dysregulation of DNA repair genes *(BRCA2, BRCA1, MSH6, PALB2, NBN*) and DNA damage sensing genes (*ATM, CHEK2, CDH1*) in IDC‐P.^4^, ^7^, ^15^ Additionally, IDC‐P tumors exhibit a higher occurrence of mismatch repair gene (MMR) aberrations (*MSH2, MSH6, MLH1, and PMS2*), despite their general rarity in prostate cancer.^16^ Prostate cancer patients with germline *BRCA2* mutations exhibit poorer survival outcomes compared to noncarriers, irrespective of whether they receive surgery or radiotherapy as radical treatment.^14^, ^72^ Risbridger et.al^14^ found that PDXs from *BRCA2*‐mutated tumors showed a higher incidence of IDC‐P compared with sporadic PCa. *BRCA2* carriers with IDC‐P had significantly worse overall and PCa‐specific survival compared with *BRCA2* carriers without IDC‐P. The data from our previous investigation^12^ also reported that the germline *BRCA2* mutation rate was markedly higher in patients with IDC‐P compared to those without IDC‐P (8.7% vs. 0%, respectively). Moreover, for mCRPC patients harboring IDC‐P components using abiraterone as first‐line systemic therapy, *BRCA2* mutation was associated with a shorter prostate‐specific antigen progression‐free survival (PSA‐PFS: median 9.1 versus 11.9 months, *p* = 0.036).

Our findings indicate that the correlation between IDC‐P and DDR mutations extends to the Chinese patient cohort, and it appears that Chinese patients with IDC‐P also exhibit a higher prevalence of *CDK12* mutations.^12^ Although *CDK12* is not traditionally considered a canonical DDR gene, it is known to influence the expression of homologous recombination repair genes.^73^, ^74^, ^75^ Despite its importance, *CDK12* is often excluded from DDR genetic testing panels in many studies regarding IDC‐P. However, patients with *CDK12* mutations tend to experience significantly worse survival outcomes and demonstrate poor response to standard treatments.^76^ Given its biological role in regulating DDR gene expression and the subsequent increase in neoantigen burden, patients with *CDK12* mutations could theoretically benefit from PARP inhibitors and immunotherapy.^77^, ^78^ Nevertheless, the existing evidence in this regard remains inconclusive.^79^, ^80^, ^81^ Therefore, it is intriguing to investigate whether a connection exists between IDC‐P and *CDK12* dysfunction.

## 5 NCOR2

Patients with IDC‐P components have been reported to be insensitive to ADT.^17^, ^18^, ^19^ The underlying reason for this phenomenon remains to be answered.

In our previous study, we observed that the mutation rate of the *AR* itself was not significantly different between tumors with and without IDC‐P.^12^ However, a noteworthy finding was the enrichment of *nuclear receptor corepressor 2 (NCOR2)* histone deacetylase in tumors with IDC‐P.^12^ While *NCOR2* was originally recognized as an AR negative regulator,^82^ emerging evidence suggests its involvement in a more complex interplay with AR signaling, influencing canonical androgen regulation and epigenetic control of enhancer regions, which may contribute to lineage plasticity changes.^20^ Reduced expression of *NCOR2* has been associated with disease aggressiveness in prostate cancer, and knockdown of NCOR2 in cell line models leads to neuroendocrine prostate cancer (NEPC) gene signatures.^20^ These findings reinforce the possible association between IDC‐P and NEPC.

Besides its role in AR signaling, *NCOR2* has been implicated in cytotoxic stress response and antitumor immunity, both of which are relevant to treatment resistance and patient outcomes in breast cancer.^83^ Loss of the transcriptional repressor function of NCOR2 results in increased expression of DUB3, which confers resistance to bromodomain and extra‐terminal (BET) inhibitors.^84^ BET inhibitors are emerging as a potential treatment for advanced prostate cancers due to their potential impact on both *MYC* expression and the *AR*.^85^

It is also important to note that the positive feedback loop between AR signaling and the DDR pathway, which can influence tumor progression and therapeutic response. Hence, while we observe the coexistence of these aberrations in tumors with IDC‐P, unraveling the driving factor for disease aggressiveness in IDC‐P or identifying the key to breaking this vicious cycle remains a significant challenge. This complexity aligns with the concept of “nimbosus,” suggesting that although we recognize these aberrations in IDC‐P, understanding their precise roles and interactions poses ongoing difficulties.^86^

## 6 MED12

RNA polymerase II (RNA Pol II) is responsible for transcribing the majority of the human genome, and this process is tightly regulated by the Mediator complex.^87^ Among the components of this complex, MED12 (Mediator complex subunit 12) plays crucial roles in various biological processes. Disruption of MED12 function has been implicated in the development of malignant diseases.^88^ Biologically, *MED12* serves as an important regulator of the Wnt pathway, encompassing both canonical Wnt signaling and Wnt/PCP signaling.^89^ A study demonstrated increased methylation levels of the *APC* gene in IDC‐P lesions, which further contributes to the activation of the Wnt pathway.^21^ Furthermore, In a study focusing on *BRCA2*‐mutant prostate cancers, it was observed that tumors with IDC‐P exhibited a higher prevalence of genomic and epigenomic dysregulation of *MED12*.^11^ Interestingly, the prevalence of *MED12* mutations varies considerably among different prostate cancer study cohorts.^22^, ^23^, ^24^ Aside from differences in patient race and sample types, we speculate that the existence of IDC‐P, as a specific pathological subtype, may also contribute to this variability. Unfortunately, the available studies have not provided data on this aspect, necessitating further investigations to address this issue. Moreover, there are conflicting views regarding the functional consequences of *MED12* mutations in prostate cancer.^22^, ^23^, ^24^ One study suggests that the tumorigenic mechanisms associated with *MED12* mutations may be cancer‐specific.^22^ Therefore, unraveling the precise workings of this network in prostate cancer could hold the key to developing effective treatments for these tumors.

## 7 TMPRSS2‐ERG FUSION

*TMPRSS2‐ERG* is a gene fusion that is commonly found in prostate cancer, which denotes the fusion of the regulatory region of the androgen‐regulated gene *TMPRSS2* to the coding region of the oncogene *ERG*. This fusion event results in the overexpression of the ERG transcription factor.^30^ It has been implicated in prostate cancer development and progression and is often used as a biomarker for this disease.

IDC‐P has been reported to exhibit a higher prevalence of *TMPRSS2‐ERG* fusions than prostatic intraepithelial neoplasia (PIN),^25^, ^26^ but the rate seems to be similar between IDC‐P and concurrent invasive carcinoma,^27^, ^28^, ^29^ and therefore a clonal relationship between IDC‐P and prostate is inferred based on this similar *TMPRSS2‐ERG* rate by some studies. *TMPRSS2‐ERG* fusion is a relatively common event seen in prostate cancer, which can be found in up to 50% of cases.^30^, ^31^, ^32^, ^33^ Therefore, the observation of *TMPRSS2‐ERG* present in both IDC‐P and PAC could be coincidental rather than indicative of a clonal relationship. To establish a definitive clonal relationship, additional molecular and genetic analyses are necessary. These analyses should encompass whole‐genome sequencing, copy number variation analysis, and the assessment of other genetic and epigenetic alterations that may be present in both IDC‐P and PAC. The relationship between the *TMPRSS2‐ERG* fusion and more aggressive tumor characteristics is currently uncertain. Some studies suggest significant associations between *TMPRSS2‐ERG* status and factors such as Gleason score, biochemical recurrence, and survival.^31^, ^34^, ^35^ However, other studies have found no significant differences related to the presence of the gene fusion.^32^, ^36^, ^37^ Interestingly, the combination of *PTEN* and *TP53* mutation status with the overexpression of *ERG* via *TMPRSS2‐ERG* fusion has shown predictive value for biochemical recurrence and survival.^38^

## 8 SCHLAP1

The second chromosome locus associated with prostate‐1 (SChLAP1) is a long noncoding RNA that has been identified in previous research as an independent predictor of unfavorable oncologic outcomes and as a potential tool for disease detection.^90^, ^91^ SChLAP1 has been reported to interfere with the tumor‐suppressive functions of the SWI/SNF complex by modulating its capacity to regulate gene expression at the post‐transcriptional level.^92^

A landscape study in IDC‐P discovered that the presence of numerous adverse molecular events in tumors with IDC‐P, including genome instability, dysregulation of SChLAP1, and hypoxia, all of which collectively contribute to the visibility on multiparametric magnetic resonance imaging and worse prognosis.^39^, ^40^ *SChLAP1* is the only gene showing up with threefold higher expression in IDC‐P among over 25,000 genes.^39^ However, as the authors also pointed out, it is possible that the presence of SChLAP1 dysregulation (also genomic instability) is not necessarily the driving force for the development of IDC‐P.^39^ Thus, it is still critical to address the chronological sequence of these molecular events.

## 9 SUMMARY AND CONCLUSIONS

Tumors containing IDC‐P display a multitude of dysregulated pathways and genes, even in the localized stage. This suggests an aggressive biological behavior and unique molecular features of IDC‐P, despite limited exposure to ADT. Given the distinct clinicopathological characteristics of IDC‐P and its significant prognostic implications, extensive research efforts have been dedicated to unraveling its molecular profile.

Further investigations are warranted to enhance our understanding of the underlying molecular landscape of IDC‐P. However, due to challenges in isolating IDC‐P from prostate cancer specimens, the genomic characteristics of IDC‐P remain largely undisclosed. One potential avenue involves employing microdissection techniques to precisely isolate specific cell populations for molecular analysis. This technique facilitates the isolation of specific components of IDC‐P, thereby minimizing confounding factors resulting from the coexistence of invasive PAC. Isolating and analyzing IDC‐P components separately provides precise insights into its molecular characteristics and unveils the intricate interplay between IDC‐P and adjacent PAC components. Furthermore, comprehensive molecular profiling of purified IDC‐P samples, including whole‐genome sequencing, transcriptomics, and epigenetic analyses, can provide valuable insights into the specific genetic and epigenetic alterations that contribute to IDC‐P's distinct phenotype. Another promising strategy involves integrating single‐cell RNA sequencing, single‐cell spatial transcriptomics, and employing artificial intelligence and deep learning methodologies. These comprehensive approaches enhance the precision of insights into the evolutionary trajectory and molecular characteristics of IDC‐P.

By understanding the specific genetic and signaling changes associated with IDC‐P, clinicians can tailor interventions to better address the distinct characteristics of this subtype of prostate cancer. This approach fosters a more personalized and targeted therapeutic paradigm, paving the way for advancements in precision medicine for individuals with IDC‐P. Further research and collaboration between clinicians and researchers are essential to unlock the full potential of these discoveries and translate them into meaningful advancements in prostate cancer care.

In summary, while considerable progress has been made in unraveling the molecular profile of IDC‐P, further investigations utilizing microdissection techniques and comprehensive molecular profiling are crucial to gain a more precise understanding of its molecular landscape. These advancements will pave the way for improved clinical management and personalized treatment approaches tailored specifically to IDC‐P patients.

## AUTHOR CONTRIBUTIONS

**Sha Zhu:** Conceptualization (equal); writing – original draft. **Nanwei Xu:** Conceptualization (equal); writing – original draft (equal). **Hao Zeng:** Supervision (equal); writing – review and editing (equal).

## FUNDING INFORMATION

This work was supported by the National Natural Science Foundation of China (NSFC 82203110, 82,172,785, and 81,974,398), 1.3.5 project for disciplines of excellence, West China Hospital, Sichuan University (ZYJC21020, ZYGD22004), Science and Technology Support Program of Sichuan Province (2021YFS0119), Clinical and Translational Medicine Research Project, Chinese Academy of Medical Sciences (2022‐I2M‐C&T‐B‐098), Bethune Foundation, Oncology Basic Research Program (X‐J‐2020‐016), and Bethune Foundation, Urological Oncology Special Research Fund (mnzl202002, mnzl202007).

## CONFLICT OF INTEREST STATEMENT

The authors declare no conflict of interest.

## Data Availability

All data mentioned in this review is available in the original article.
